# Integration of *in vitro* and *in silico* Models Using Bayesian Optimization With an Application to Stochastic Modeling of Mesenchymal 3D Cell Migration

**DOI:** 10.3389/fphys.2018.01246

**Published:** 2018-09-11

**Authors:** Francisco Merino-Casallo, Maria J. Gomez-Benito, Yago Juste-Lanas, Ruben Martinez-Cantin, Jose M. Garcia-Aznar

**Affiliations:** ^1^Multiscale in Mechanical and Biological Engineering, Department of Mechanical Engineering, Aragón Institute of Engineering Research, Universidad de Zaragoza, Zaragoza, Spain; ^2^Centro Universitario de la Defensa, Zaragoza, Spain; ^3^SigOpt, Inc., San Francisco, CA, United States

**Keywords:** 3D mesenchymal migration, fibroblast, chemotaxis, platelet derived growth factor, phosphoinositide 3-kinase, tau-leaping algorithm, Bayesian optimization, *in-vitro in-silico* integration

## Abstract

Cellular migration plays a crucial role in many aspects of life and development. In this paper, we propose a computational model of 3D migration that is solved by means of the tau-leaping algorithm and whose parameters have been calibrated using Bayesian optimization. Our main focus is two-fold: to optimize the numerical performance of the mechano-chemical model as well as to automate the calibration process of *in silico* models using Bayesian optimization. The presented mechano-chemical model allows us to simulate the stochastic behavior of our chemically reacting system in combination with mechanical constraints due to the surrounding collagen-based matrix. This numerical model has been used to simulate fibroblast migration. Moreover, we have performed *in vitro* analysis of migrating fibroblasts embedded in 3D collagen-based fibrous matrices (2 mg/ml). These *in vitro* experiments have been performed with the main objective of calibrating our model. Nine model parameters have been calibrated testing 300 different parametrizations using a completely automatic approach. Two competing evaluation metrics based on the Bhattacharyya coefficient have been defined in order to fit the model parameters. These metrics evaluate how accurately the *in silico* model is replicating *in vitro* measurements regarding the two main variables quantified in the experimental data (number of protrusions and the length of the longest protrusion). The selection of an optimal parametrization is based on the balance between the defined evaluation metrics. Results show how the calibrated model is able to predict the main features observed in the *in vitro* experiments.

## Introduction

Cell migration is a fundamental event in a wide variety of physiological processes, spanning from embryogenesis (Knecht and Bronner-Fraser, 2002; Martin and Parkhurst, 2004), angiogenesis (Lamalice et al., 2007; Spill et al., 2015), osteogenesis (Reina-Romo et al., 2012), inflammatory response (Luster et al., 2005), immune response (Bogle and Dunbar, 2010), and wound healing (Shaw and Martin, 2009; Valero et al., 2014a), to develop diseases such as cancer and metastasis formation (Franz et al., 2002; Condeelis et al., 2005; Condeelis and Pollard, 2006).

Cell migration can present different characteristics according to the dimensionality in which it is produced. Thus, cell migration on 2D surfaces has been widely studied and is typically characterized by a balance between counteracting traction and adhesion forces (Sunyer et al., 2016; Escribano et al., 2018). However, cells generally migrate in a 3D extracellular matrix (*ECM*) adopting different migration strategies regulated by several factors such as the cell type and the properties of the ECM. In these 3D environments, the mechanisms governing cell migration are far less understood due to both the technical challenges and the complexity of migratory behaviors (Zhu and Mogilner, 2016).

Based on the cell type and the cellular microenvironment (Te Boekhorst et al., 2016; Talkenberger et al., 2017)—in particular ECM parameters such as density, porosity and stiffness—, individual cells migrate using two distinct mechanisms (Friedl and Wolf, 2010; Swaney et al., 2010; Bear and Haugh, 2014). When cells are unable to adhere to the ECM, they modify their shape and squeeze through the ECM pores by using the amoeboid migration, which is very efficient—rapid cell locomotion (cell speed ~10 μm/min)—and it is observed in cells such as neutrophils and T cells (immune system) (Beauchemin et al., 2007; Lämmermann et al., 2008; Swaney et al., 2010). In contrast, whenever cells adhesion to the ECM is high, they degrade the ECM to pass through by using the mesenchymal migration mode, which is very inefficient—cell displacement is very slow (cell speed < 1 μm/min)—and it is observed in cells such as fibroblast (wound healing) and osteoblasts (bone formation) (Friedl and Wolf, 2010). This mesenchymal migration mode is investigated in this paper.

*In vitro* experiments have become increasingly sophisticated in order to reproduce as accurate as possible the natural biological surroundings of organisms from *in vivo* studies. As *in vitro* studies have increased their sophistication, their requirements have also grown in complexity. Due to the complexity and the expensive lab work of *in vitro* experiments, *in silico* studies have a complementary role in understanding mesenchymal cell migration. Computer-based mathematical models allow performing a vast number of controlled and reproducible experiments with much lower associated costs. In fact, these computational models can be classified according to several factors such as the numerical approach of the biological processes: continuous (Vermolen and Javierre, 2012; Valero et al., 2014b; Serrano-Alcalde et al., 2017), discrete (Meineke et al., 2001; Bentley et al., 2009; Scianna et al., 2012; Scianna and Preziosi, 2014; Van Liedekerke et al., 2015), or hybrid (Alber et al., 2007; Bauer et al., 2009; Daub and Merks, 2013; Milde et al., 2014; González-Valverde and García-Aznar, 2018). In addition, they can also be classified according to the type of movement that cells develop as individual (Schlüter et al., 2012; Trichet et al., 2012; Ribeiro et al., 2017; Moure and Gomez, 2018), if cells migrate independently, or collective, forming an interconnected meshwork or cluster (Bazmara et al., 2015; González-Valverde et al., 2016; Norton and Popel, 2016; Camley and Rappel, 2017; Escribano et al., 2018). Computational models can also be classified as mechanical (Zaman et al., 2005; Borau et al., 2011), biochemical (Hatakeyama et al., 2003; Provenzano et al., 2008), or mechano-chemical (Kim et al., 2015, 2018; Moure and Gomez, 2017; Ribeiro et al., 2017).

More recently, different authors (Sun and Zaman, 2017; Kim et al., 2018; Mark et al., 2018) have focused their works in the combination of *in vitro* experiments and *in silico* modeling in order to elucidate the influence of specific factors on individual and collective cell migration. The combination of both methodologies opens new opportunities for research, because models allow the simulation of *in vitro* conditions in order to directly obtain additional information not available from experiments, but that can be indirectly evaluated *in-vitro*. For example, recently, Sunyer et al. (2016) analyzed collective cell durotaxis, combining experiments with numerical models in order to understand that the difference of stiffness sensed by cells at both edges of the cell monolayer promotes the directional migration.

In this work, we propose to establish a new strategy based on the Bayesian optimization (*BO*) technique, which combines numerical simulations relied on a mathematical model and *in vitro* experiments in order to calibrate the model's parameters. In particular, a mechano-chemical model of individual mesenchymal 3D migration is presented, with a focus on accelerating the numerical simulations that determine the 3D migration trajectories. This strategy allows the full integration of numerical models and experimental measurements in order to improve knowledge of how cells regulate this mesenchymal 3D migration.

## Materials and methods

This section is organized in order to describe how experimental measurements and numerical simulations can be integrated in a consistent way. To facilitate their explanation, first, we briefly describe the mathematical model of cell migration (Ribeiro et al., 2017) and its numerical implementation. Next, we show the results from *in-vitro* experiments and their quantification. Then, we present how both results can be combined by means of Bayesian optimization in order to calibrate the numerical model with the experimental results. Finally, we test the potential of our calibrated numerical model under different chemoattractant concentrations and gradients.

### Model description

**Figure 1:**
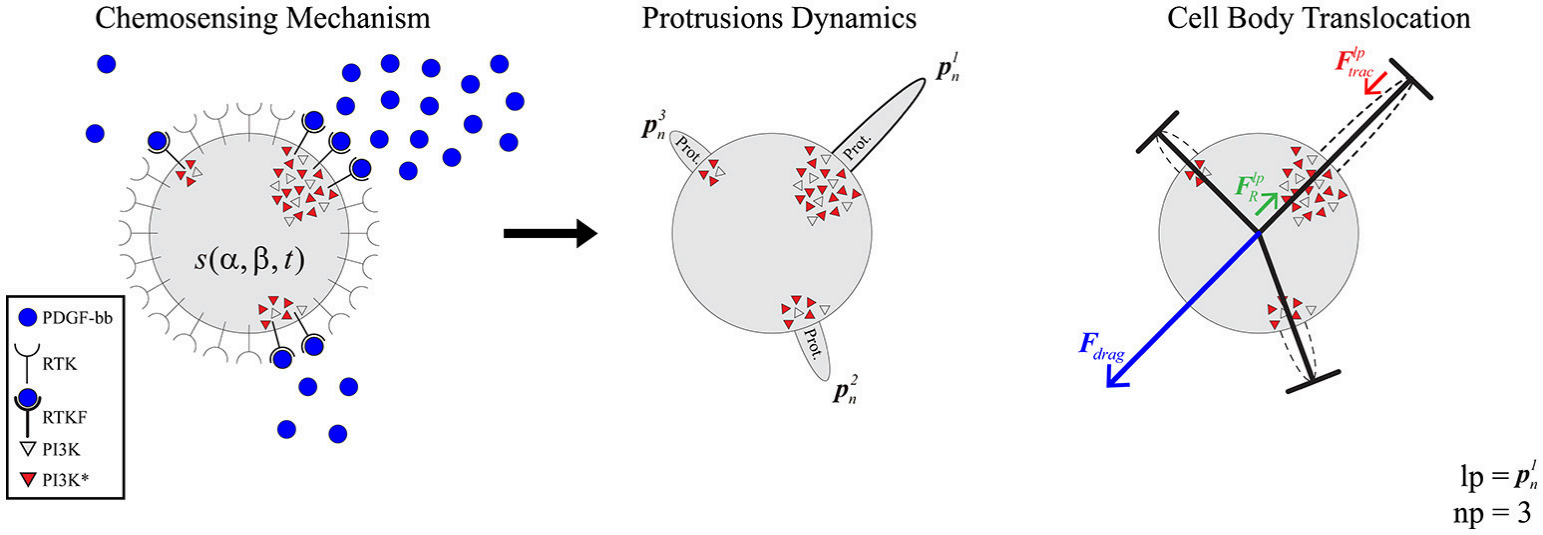
Global model scheme. **(Left)** the chemosensing mechanism simulates how RTKs located in the cell membrane become activated by binding to PDGF-BB molecules (blue circles). This RTK activation, in turn, triggers the activation of PI3K molecules inside the cell (PI3K inactivated molecules as gray triangles and PI3K activated ones as red triangles). **(Middle)** protrusions (${\text{p}}_{n}^{i}$) grow and stabilize on those areas with high concentration of PI3K activated molecules. **(Right)** the longest protrusion generates a traction force (${\text{F}}_{trac}^{lp}$) when retracting, which exerts a reaction force (${\text{F}}_{R}^{lp}$) over the cell body. As a result of these reaction forces, the ECM generates a drag force (**F**_*drag*_) over the cell body.

The selected model to simulate 3D cell migration is based on a previous one (Ribeiro et al., 2017) (Figure 1). Here, we describe the main aspects of this model in order to understand how the full calibration of this model is developed. This model assumes that cell migration can be described by three clearly differentiated stages. During the first stage the cellular chemosensing mechanism allows cells to probe the chemical cues located on their surroundings through different membrane receptors (Roca-Cusachs et al., 2013; Moreno-Arotzena et al., 2015). In particular, the focus is on how fibroblasts detect molecules of the chemo-attractant factor Platelet-derived Growth Factor (*PDGF* from now on) through a specific cell surface receptor, the tyrosine kinases one (also known as *RTK*) (Cao et al., 2004; Poukkula et al., 2011). The second stage simulates how the activation of these receptors triggers intracellular processes that regulate the onset of dendritic protrusions in different directions throughout the ECM (Weiger et al., 2010; Liou et al., 2014). In fact, these protrusions can protrude (pushing the matrix) and contract (pulling the matrix). Lastly, the third stage models how the dynamics of these protrusions regulate cell migration in 3D (Campellone and Welch, 2010; Starke et al., 2013; Moreno-Arotzena et al., 2014) by establishing a relation between the contractile force generated by each protrusion and the cell body translocation.

Next, these three main stages of the process of cell migration are described in greater detail. But first, the model of 3D cell behavior is defined.

#### Modeling cell behavior

The 3D structure of the cell is geometrically modeled as a set of one-dimensional bars representing dendritic protrusions (Ribeiro et al., 2017). Those bars are located in a three-dimensional environment and diverge from a central connecting point that represents the cell body. This central connecting point—which can be associated to the cell nucleus or the cell centrosome—exists solely for modeling purposes as the point where all the bars are connected (Figure 1 right).

#### Modeling the chemosensing mechanism

This first stage models how the chemically reacting system that allows the cell to sense the chemo-attractant factor (located in the surrounding ECM) evolves through time (Figure 1 left).

It is assumed that the only signal pathway guiding protrusion dynamics is the one including just a chemo-attractant factor located in the ECM, RTKs in the cell membrane and PI3K molecules inside the cellular body. The PDGF has been chosen as the chemical factor to interact with the cell due to its pivotal role in regenerative processes (Chen et al., 2007; Friedlaender et al., 2013; Elangovan et al., 2014; Shah et al., 2014). However, the model could be extrapolated to other growth factors.

In order to replicate the cellular chemosensing mechanism, our model simulates the interaction between different species through time. From a temporal perspective, the simplified mathematical model that mimics this chemosensing mechanism is based on a set of reactions (Equation 1) (Hatakeyama et al., 2003; Hawkins et al., 2006) and defined by a set of differential equations (Equation *S*1 of the Supplementary Material).

(1) $$\{\begin{array}{c} \;RTK+{\left[F\right]}_{k_{2}}{⇌}^{k_{1}}RTKF\text{ ​​ }(R_{1}\;R_{2})\;\\ RTKF+PI3K\to_{k_{3}}RTKF+PI3K_{A}\;\left(R_{3}\right)\;\\ PI3K_{A}\to_{k_{4}}PI3K\;(R_{4})\; \end{array}$$

From a spatial perspective, it is assumed that membrane receptors such as RTKs are homogeneously distributed over the cell surface. However, the activation density of such membrane receptors depends on the distribution of chemoattractant molecules (*F*). In particular, there are more activated RTK receptors (*RTKF*) on those areas of the cell surface surrounded by a higher concentration [*F*]. In contrast, on those areas of the cell surface surrounded by a lower concentration [*F*], there are less activated receptors. Thus, cells are able to sense the spatial distribution of *F*.

Two sources of stochasticity in cell migration are associated to the chemosensing mechanism: the evolution of the chemically reacting system (defined by Equation 1 for the proposed model) and the activation of RTKs based on the concentration of chemoattractant molecules surrounding the cell. Therefore, the chemical reactions defined by Equation (1) are assumed to be stochastic processes described by a Poisson distribution (Ueda and Shibata, 2007). This premise makes possible to consider receptor activation over a domain with varying concentration of factor *F* to be a multivariate non-homogeneous Poisson's distribution. Therefore, it is possible to model this activation of RTKs by means of the Inverse Method described by Saltzman et al. (2012).

**Figure 2:**
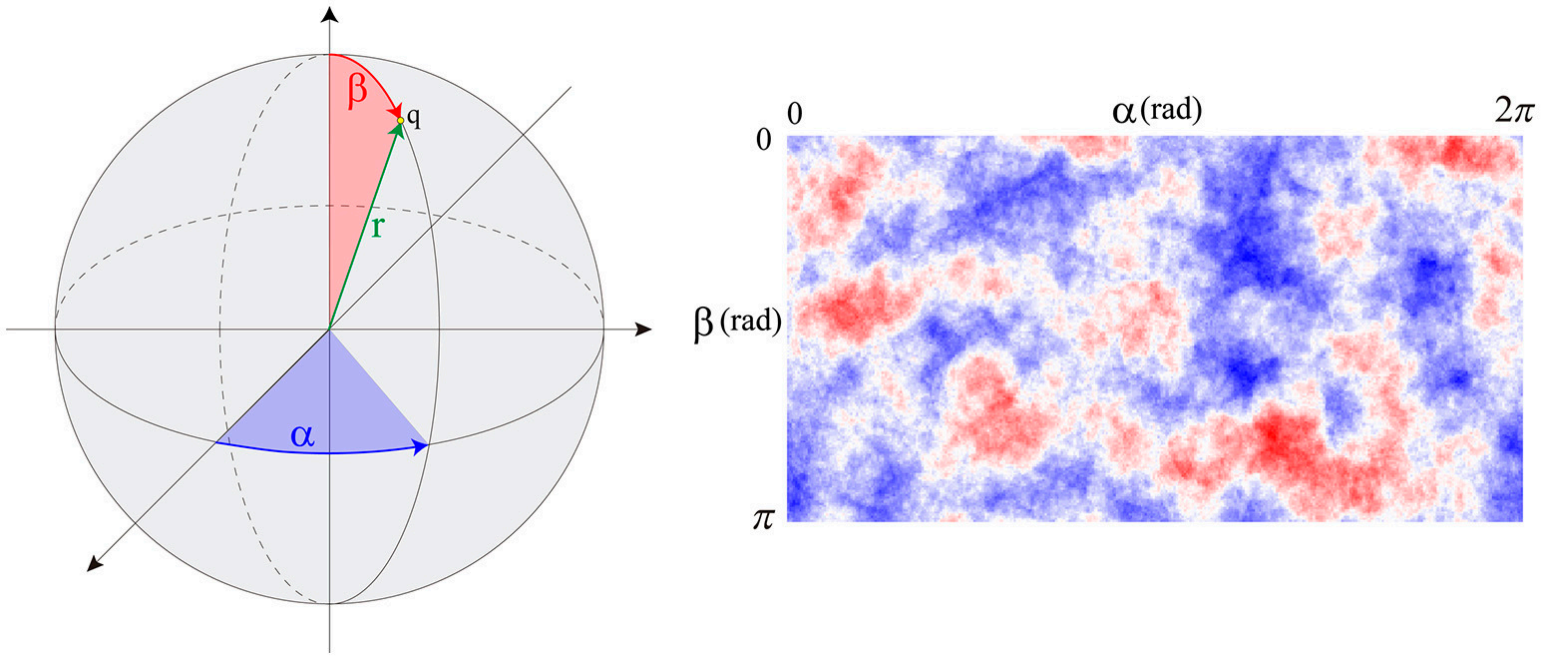
**(Left)** spherical coordinates (*α*, *β*) of point *q*. **(Right)** s signal distribution example over the cell membrane.

By computing an approximate solution of this problem, it is possible to evaluate at any given time (*t*_*k*_) the variation of PI3K_A_ in any specific location of the cell surface. In order to estimate this spatio-temporal variation we defined the variable *s*(*α*, *β*, *t*_*k*_) which stores the spatial persistence of PI3K_A_ activation across time (*t*_*k*_), in a space location of the cell surface defined by coordinates (*α*, *β*) (Figure 2)—since we are dealing with a 3D model of a cell, we represent the cell membrane as a flat surface defined by the polar coordinates *α* and *β*. Therefore, the signal *s* = *s*(*α*, *β*, *t*_*k*_) is evaluated at a fixed time *t*_*k*_ by means of the convolution function, taking into account the temporal evolution of the chemical signal in this surface location and its surroundings—roughly an area the size of a protrusion section. The model equations guiding the chemosensing mechanism are summarized in Table 1.

**Table 1:** Equations associated with the chemosensing mechanism.

**Spatial persistence of PI3K_A_ (2D convolution over the cell surface)**	**$s_{t}\left(\alpha ,\;\beta \right)=\overset{+\infty }{\underset{-\infty }{\int }}\overset{+\infty }{\underset{-\infty }{\int }}d_{{PI3K}_{A}}\left(u,\;v\right)\cdot g\left(\alpha -u,\beta -v\right)dudv$**	**(Equation 2)**
Tempo-Spatial variation of PI3K_A_	$s=s\left(\alpha ,\;\beta ,t_{k}\right)=\overset{t_{k}}{\underset{{t=t}_{0}}{\sum }}s_{t}\left(\alpha ,\;\beta \right)$	(Equation 3)

*s_t_ (*α*, *β*): amount of activated PI3K (PI3K_A_) across the cell membrane surface at a time t*.

*d_PI3K_A__(*α*, *β*): distribution of PI3K_A_ molecules across the membrane*.

*g(*α*, *β*): a convolution window roughly the size of a protrusion*.

*t_0_, t_k_: time period through which s_t_ (*α*, *β*) is sampled at 1 Hz and evaluated at 5-min intervals*.

*s(*α*, *β*, t_k_): the cumulative signal of PI3K_A_ (s from now on) at time t_k_*.

We assume that cell's consumption of chemoattractants is negligible. Thus, the chemoattractant chemical profile does not change with time.

#### Modeling protrusion dynamics

Once the tempo-spatial variation of activated PI3K (PI3K_A_, *s*) is estimated, it is possible to determine protrusions location by means of a set of thresholds (*s*_*birth*_, *s*_*exp*_, *and s*_*ret*_) that act as a signal filter. In particular, *s*_*birth*_ represents the minimal amount of signal *s* that cells need to develop new protrusions, as suggested by many authors (Ueda and Shibata, 2007; Weiger et al., 2010; Jilkine and Edelstein-Keshet, 2011; Chen et al., 2017); those points inside the cellular body where *s* is higher than *s*_*birth*_ are considered locations where novel protrusions sprout. Furthermore, any pre-existing protrusion becomes reinforced if, in its location, *s* is higher than *s*_*exp*_. However, if there is not enough signal *s* for the protrusion to remain active, it becomes unstable; in those points where *s* is lower than *s*_*ret*_ pre-existing protrusions retract and disappear (Table 2). Besides, in order to simplify the search of signal *s* peaks where protrusions centroids are localized, an internal model parameter (*s*_*binary*_) is used to transform *s* into a binary signal. This means that only during the protrusions localization, any surface point where *s* is lower than *s*_*binary*_ becomes 0 whereas every surface point with *s* greater or equal to *s*_*binary*_ becomes 1.

**Table 2:** Equations associated with the protrusions dynamics.

**Protrusion *i***	**$p_{n}^{i}=|p_{n}^{i}|e_{i}$**	**(Equation 4)**
Free *exp*./*ret*. (usions deformation)	$\varepsilon_{k}^{f}=\{\begin{array}{c} \;\frac{1}{\left\|\;p^{i}\right\|}\frac{\alpha_{exp}\;\delta_{s}}{\left(\beta_{exp}+\delta_{s}\right)},\;\delta_{s}\geq 0\;\left(expansion\right)\\ \frac{1}{\left\|\;p^{i}\right\|}\frac{\alpha_{ret}\delta_{s}}{\left(\beta_{ret}+\delta_{s}\right)},\;\delta_{s}<0\;\left(retraction\right) \end{array}$	(Equation 5)
Free *exp*./*ret*. Cauchy's strain tensor	$\varepsilon_{k}^{f}=\varepsilon_{k}^{f}e_{i}⊗e_{i}$	(Equation 6)
Constrained *exp*./*ret*. by the ECM	${\tilde{\varepsilon }}_{k}^{c}=S{\left[\left(C_{I}-C_{M}\right)\;S+C_{M}\right]}^{-1}C_{I}\;{\tilde{\varepsilon }}_{k}^{f}\left(Eshelby^{\prime }s\;theory\right)$	(Equation 7)
Constrained *exp*./*ret*. (ECM does restrict protrusions deformation)	$\varepsilon^{EXP}=\{\begin{array}{c} \varepsilon_{exp}^{c},\;\left(\left\|p_{n}^{i}\right\|=0\right)\;and\;(s_{birth}<s)\\ 0,\;\left(\left\|p_{n}^{i}\right\|=0\right)\;and\;(s_{birth}\geq s)\\ \varepsilon_{exp}^{c},\;\left(\left\|p_{n}^{i}\right\|>0\right)\;and\;(s_{exp}<s)\\ 0,\;\left(\left\|p_{n}^{i}\right\|>0\right)\;and\;(s_{exp}\geq s) \end{array}\;$ $\varepsilon^{RET}=\{\begin{array}{cc} \varepsilon_{ret}^{c}, & s_{ret}\leq s\\ -1, & \;s_{ret}>s \end{array}$ Where $\varepsilon_{k}^{c}$ comes from the inverse of Equation 6 after computing the constrained *exp./ret*. of Equation 7.	(Equation 8)
Protrusion length	$p_{n+1}^{i}=\left({1+\varepsilon }^{EXP}+\varepsilon^{RET}\right)|p_{n}^{i}|e_{i}$	(Equation 9)

*$p_{n}^{i}$: protrusion i at time interval n*.

*$\left||p_{n}^{i}|\right|$: length of protrusion i at time interval n*.

***e**_i_: unit vector of the protrusion longitudinal axis*.

*k: exp (expansion), ret (retraction)*.

**Figure 3:**
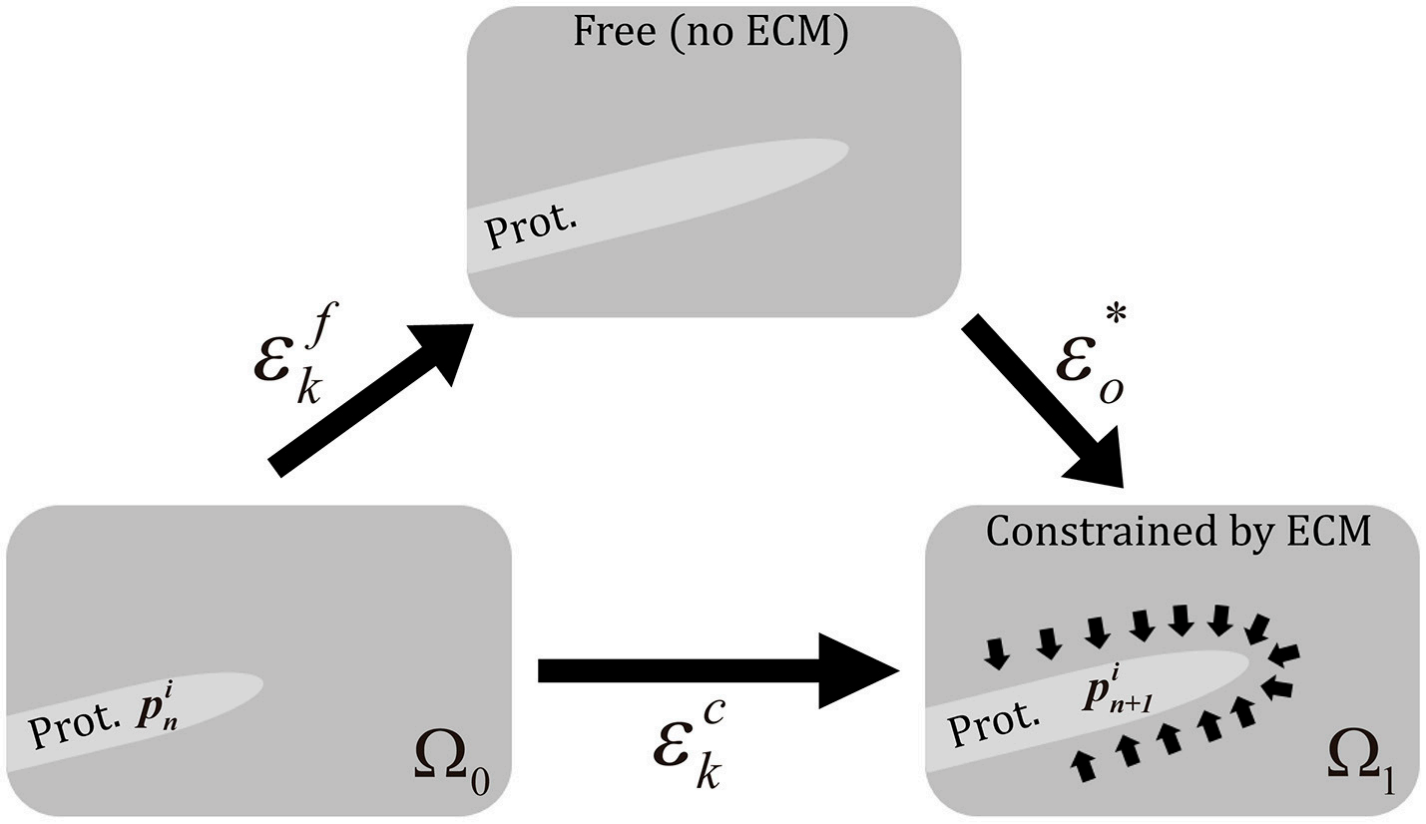
Scheme of the three different configurations in protrusion dynamics. $\varepsilon_{k}^{f}$ represents the free expansion/retraction (ECM does not restrict protrusions deformation) Cauchy's strain tensor. **ε**_o_* is the compatibility Cauchy's strain tensor. $\varepsilon_{k}^{c}$ represents the total deformation Cauchy's strain tensor. We assume infinitesimal deformation.

*$\varepsilon_{k}^{f}$: free expansion/retraction stretch rate field (see Figure 3)*.

*α_exp_, *β*_exp_, *α*_ret_, *β*_ret_: parameters that regulate protrusion expansion/retraction*.

*δ_s_: increment in signal s between two time instants*.

***S**: ellipsoid shape tensor*.

***C**_I_: elasticity tensor of the protrusion*.

***C**_M_: elasticity tensor of the surrounding ECM*.

*${\tilde{\varepsilon }}_{k}^{f}$: Cauchy's strain tensor of protrusion free expansion or retraction (see Figure 3)*.

*$\varepsilon_{k}^{c}$: constrained expansion/retraction stretch rate field*.

*ε^EXP^: longitudinal stretch rate due to the dendritic expansion*.

*Both ${\tilde{\varepsilon }}_{k}^{c}$ and ${\tilde{\varepsilon }}_{k}^{f}$ are in Voigt notation and represent the second-order stretch tensors $\varepsilon_{k}^{c}$ and $\varepsilon_{k}^{f}$ respectively. For this approach, it is considered a linear elastic behavior for the ECM*.

In addition, it is assumed that this signal variation δ*s* also regulates, in conjunction with the ECM mechanical properties, the protrusive stretch characteristics due to the cytoskeleton activity.

Therefore, protrusions generate forces against the ECM. Consequently, the mechanical properties of the ECM act as a regulator for the extension or retraction of protrusions, as suggested by Liou et al. (2014) (Figure 3). This behavior is simulated by considering protrusions analogous to an elastic inclusion (ellipsoid) embedded in the ECM, applying Eshelby's analytical solution of ellipsoidal elastic inclusions in an elastic infinite body (Eshelby, 1957). We consider that during this second stage protrusions grow inside a collagen-based fibrous matrix and they adhere to ECM fibers. Thus, we consider the ECM behaves as a linear elastic material that constrains the growth of protrusions. In fact, during this growth, protrusions push to the ECM deforming it and the elastic properties of the ECM regulate this deformation. In this case we quantify the growth of the protrusion and the deformation of the ECM by means of the Eshelby's theory, assuming the protrusion as an inclusion that is embedded in the ECM. Moreover, in all the cases we assume infinitesimal deformation.

Equations guiding protrusions dynamics are summarized in Table 2.

#### Modeling cell body translocation

Finally, based on the experimental observations of how protrusions determine cell body translocation (Moreno-Arotzena et al., 2015; Del Amo et al., 2017; Movilla et al., 2018), it is assumed that the longest protrusion determines cell motion directly. The longest protrusion presents a larger adhesion surface and, consequently, adhesion proteins have higher probability to connect with the ECM. Every cell protrusion, except the leading one, becomes non-adherent and, as a result, they are all dragged by the cell during cell motion. The retraction of the leading protrusion generates a reaction force ($F_{R}^{lp}$) supported by the cell body. Thus, by focusing just on the reaction force generated by the longest protrusion ($F_{R}^{lp}$), it is possible to estimate the exerted drag force (***F***_*drag*_) by the ECM on the cell body (Figure 1 right). As a result, both cell speed and position can be estimated at any given time *t* following the definition proposed by Borau et al. (2011), which takes into account the ECM viscosity. During the third stage we model the cell body translocation and, as the position of the cell center is modified, we assume the cell body is on the fluid component of the ECM. Thus, we consider that the cell is moving through a fluid. As a result, and in order to compute the drag force exerted by the ECM on the cell body, we take into account the viscosity of the ECM. The equations guiding cell body translocation are summarized in Table 3.

**Table 3:** Equations associated with the body translocation.

**Force equilibrium equation**	**$F_{drag}+F_{R}^{lp}=0$**	**(Equation 10)**
Drag force exerted by the ECM on the cell body	***F***_*drag*_ = −6π*rη****v***	(Equation 11)
Contractile force generated by each protrusion	$F_{trac}^{i}={-\alpha }_{adhesion}\cdot p^{i}=-F_{R}^{i}$	(Equation 12)
Final force equilibrium equation	$-6\pi r\eta v+\alpha_{adhesion}\cdot p^{lp}=0$	(Equation 13)

***F**_drag_: drag force exerted by the ECM on the cell body*.

*$F_{R}^{lp}$: reaction force supported by the cell body due to the retraction force of the longest protrusion (lp)*.

*r: cell radius*.

*η: ECM viscosity*.

***v**: cell speed*.

*$F_{trac}^{i}$: contractile force of protrusion i*.

**α*_adhesion_: constant that defines adhesion*.

***p**^i^: vector representing the protrusion i*.

***p**^lp^: vector representing the longest protrusion*.

*Traction forces $F_{trac}^{i}$ are assumed identical in magnitude to their corresponding reaction forces $F_{R}^{i}$*.

We assume that there is a mechanical balance between the traction force of the adherent protrusion ($F_{trac}^{lp}$), the longest one, and its corresponding reaction force ($F_{R}^{lp}$) supported by the cell body due to $F_{trac}^{lp}$ (Figure 1 right). Equation (12) defines a relationship between the contractile force magnitude ($F_{trac}^{lp}$), due to actomyosin activity, and the protrusion length.

### Numerical implementation

**Figure 4:**
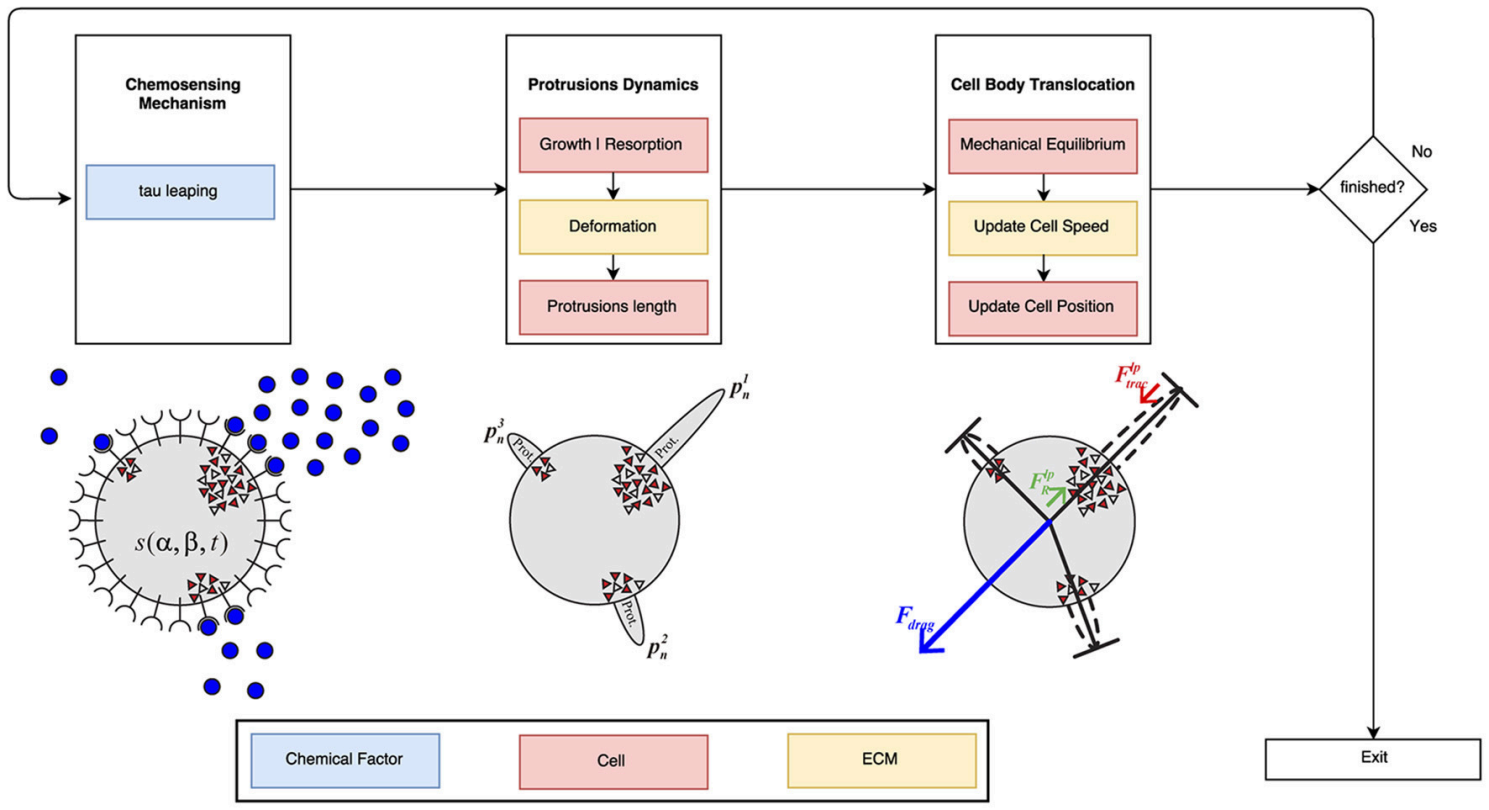
Global algorithm scheme. First, the chemosensing mechanism is simulated using the tau leaping algorithm. In this first stage, the concentration and gradient of the PDGF-BB is the main influence factor. During the second stage of the process, it is taken into account both ECM mechanical properties and cell mechanics in order to simulate protrusions development. Finally, the modeling of the cell body translocation is also influenced by the ECM mechanical properties (in particular, ECM viscosity) as well as cell mechanics. The blue boxes are associated with the chemical factor, the red ones with cell mechanics and the yellow ones with the ECM mechanical properties.

Our computational model has been designed using a scheme based on the three fundamental mechanisms: chemosensing mechanism, protrusions dynamics, and the cell body translocation (Figure 4). These three stages have been implemented in Python using powerful packages and libraries for scientific computing such as Numpy (van der Walt et al., 2011) and SciPy (Jones et al., 2001) to maximize the model's performance.

The stochastic time evolution of the given set of reactions (*R*_1_, *R*_2_, *R*_3_, *and R*_4_) had been numerically simulated by using, originally, the Stochastic Simulation Algorithm (*SSA*; also known as the Gillespie Algorithm) (Gillespie, 1976, 1977) in the first version of this work (Ribeiro et al., 2017). However, the SSA is considered too slow for our purposes and a faster alternative is proposed, the tau-leaping algorithm (Gillespie, 2001; Cao et al., 2006). The SSA computes an exact solution of the time evolution of a chemically reacting system. In contrast, the tau-leaping algorithm estimates a good enough^1^ approximation (Lok, 2004; Cazzaniga et al., 2006) by leaping over many reactions at once using Poisson random numbers.

The tau-leaping method tries to accelerate stochastic simulations by approximating the frequency of each reaction being fired in the next specified time interval [t, t+τ]. By comparison, the SSA focuses only on one reaction per time interval which may be prohibitively small (Anderson et al., 2011). As long as the value of τ is small enough so the leap condition^2^ is satisfied, it is possible to compute a good approximation of the evolution of a given chemically reacting system.

It is worth to mention that neither the SSA nor the tau-leaping algorithm use a fixed time step to simulate the evolution of biologically reacting systems like the one presented in this work. Instead, they compute a new value τ in each iteration based on the current state of the system and a random variable.

The initial amounts of each reactant as well as the reaction rates (*k*_1_, *k*_2_, *k*_3_, and *k*_4_) used are included in Table 4.

**Table 4:** Initial amounts of each reactant as well as the reaction rates obtained from literature.

**Reactant**	**Initial amount**	**Equation**	**References**
*RTK*	4275	(1)	Paralkar et al., 1992
*RTKF*	0	(1)	Estimated
*PI*3*K*	75 *x* 10^3^	(1)	Hatakeyama et al., 2003
*PI*3*K*_*A*_	0	(1)	Estimated
*k*_1_	735 *nM*^−1^·*s*^−1^	(1)	Heinecke et al., 2009
*k*_2_	0.01 *s*^−1^	(1)	Heinecke et al., 2009
*k*_3_	0.0004 *s*^−1^	(1)	Hatakeyama et al., 2003
*k*_4_	1 *s*^−1^	(1)	Hatakeyama et al., 2003

Based on the spatial distribution of PI3K_A_ molecules as well as their concentration on those locations, protrusion growth is then set. Protrusion final length is computed by applying Eshelby's solution of ellipsoidal elastic inclusions in an infinite elastic body. Mechanical equations are analytically solved using a computational algorithm. An elastic modulus of 104 Pa is assumed for the ECM based on previous experimental works of gels with a concentration of 2 mg/ml collagen type I (Movilla et al., 2018; Valero et al., 2018).

Lastly, the mechanical equilibrium associated to protrusion-generated forces is solved. Then, taking into account that the longest protrusion is the one leading cell migration, it is computed both cell speed and position in the following time increment.

We decouple the simulation of the chemosensing mechanism from the other two stages of the model (protrusions dynamics and cell body translocation) because we are considering two different time scales in our model. In fact, the chemical and mechanical events occur at different time scales. In order to accurately simulate the proposed chemically reacting system we are using the iterative tau leaping algorithm with a variable associated time step τ in the range [0.5, 1.5] seconds. However, to model protrusion dynamics and the cell body translocation we are using a time step *dt* of 5 min. Actually, it is because variations in the signal *s* (Equation 2) between two consecutive time steps *t* and *t*+τ are really small whereas protrusions require more noticeable variations of the chemical signal in order to change their current state. As a result, it is required to keep track of the cumulative variation δ*s*.

### Development and quantification of *in vitro* experiments

Once we have numerically implemented the proposed model, we have to calibrate its parameters in order to optimize the performance of this computational model. We calibrate the model here presented by comparing the results of its simulations with experimental data. In particular, we focus on two different features to fit the model's parameters: the length of the longest protrusion and the number of protrusions of the migrating cell. As a result, we have performed *in vitro* studies to get accurate experimental measurements of the length of the longest protrusion and the number of protrusions.

**Figure 5:**
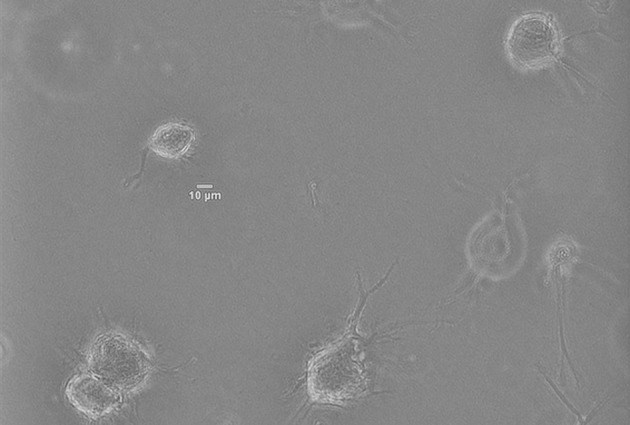
Norman Human Dermal Fibroblast (NHDF) cultured in 3D collagen-based fibrous matrix (2 mg/ml collagen). Image was captured with a Nikon D-Eclipse Microscope with a Plan Fluor 200x magnification (20x Objective) and phase contrast.

*In vitro* experiments have been performed by culturing Normal Human Dermal Fibroblasts (NHDF)—human skin primary cells—within 2 mg/ml collagen gels at a concentration of 2.5x10^5^ cells/ml, and temperature and atmosphere conditions have been maintained at 37°C and 5% CO_2_. Immediately after the seeding, cells' evolution has been monitored with multidimensional microscopy for 4 h (from 0 to 4 h), every 5 min and 5 μm of Z axis, with 200x magnification (20x objective) and phase contrast (Figure 5). We have chosen a 2 mg/ml collagen concentration because it already implies a matrix pore size (< 1 μm) (Fraley et al., 2010). Individual cell protrusions have been quantified by in-house Matlab algorithms (Moreno-Arotzena et al., 2015). For each image stack, best Z has been chosen in order to maximize accuracy and minimize the complexity of the manual analysis of both the cell body and its protrusions. Single cell analysis of four different samples has been performed for the given collagen concentration (2 mg/ml).

FGM™-2 (Fibroblast Growth Medium-2) has been used to support the growth of primary human fibroblasts. It contains a supplementation of GA-1000, recombinant human insulin 0.5%, HFGF-B GF, and 2% of Fetal Bovine Serum. Thus, these *in vitro* experiments only include a very low and fixed concentration of growth factors included in the culture medium; they do not include any chemoattractant gradient.

### Model calibration using bayesian optimization

During the last couple of decades, as the available computational power has greatly increased, so has the complexity of *in silico* models and the number of parameters included in those models. As a result, the complexity of the calibration process has also increased. However, it is still often the case that this calibration process is performed using a very manual approach. Each parameter must be tuned manually despite the search space being usually too vast to be effectively navigated. Besides, there may be interactions or dependencies between some parameters. This process can be very tedious, especially when dealing with *in silico* simulations that require several hours of execution time.

This calibration process can be mapped to a non-linear optimization problem where the objective is to find the simulation parameters that best fits the *in vitro* experiments. In this way, we are able to automate the process. However, most non-linear optimization solvers require a large number of iterations, gradient information of the fitting function or they are sensible to local optima. In our case, the large number of iterations could make the problem intractable as the evaluation of the fitting function associated to our *in silico* model is very costly because it requires several simulations of our stochastic model.

More formally, we are looking for the set of optimal experiment parameters *x* that satisfy:

(14) $$\begin{array}{r} x^{⋆}=\underset{x\in \chi }{\text{argmax}}f\left(x\right), \end{array}$$

where *f* is the fitting function between the *in vitro* and the *in silico* models and X is the parameter search space as defined in Table 5.

**Table 5:** Model parameters calibrated using Bayesian optimization with SigOpt.

**Parameter**	**Calibrated value**	**Equation**	**Range**
*E*_*protrusion*_	10^7^ *Pa*	(7)	10^*i*^, *i*∈{4, 5, …, 10}
*s*_*birth*_	85	(9)	ℤ∈[0, 100]
*s*_*exp*_	76	(9)	ℤ∈[0, 100]
*s*_*ret*_	0	(9)	ℤ∈[0, 100]
*α*_*exp*_	0.14 *mm*	(7)	ℝ∈[0.01, 0.2]
*β*_*exp*_	100	(7)	ℝ∈[0.1, 100]
*α*_*ret*_	0.05 *mm*	(7)	ℝ∈[0.01, 0.2]
*β*_*ret*_	54.86	(7)	ℝ∈[0.1, 100]
*s*_*binary*_	62500		12500+2000·*j, j*∈{0, 1, …, 100}

*The calibrated values are associated to the parametrization considered the best one (with computed metrics BC_llp_ = 0.87 and BC_np_ = 0.81). The given ranges have been established at the beginning of the calibration process and leave them unchanged*.

Bayesian optimization, also called Efficient Global Optimization (EGO) (Jones et al., 1998) is a general purpose black-box optimization methodology that it is characterized for requiring a very small number of iterations before reaching global optimization. Thus, it is especially suitable for experimental design and calibration of expensive processes (Shahriari et al., 2016). Bayesian optimization uses a probabilistic surrogate model of the target function combined with optimal decision theory to drive the search toward the global optimum in less iterations than popular non-linear optimization alternatives like PSO (Kennedy and Eberhart, 1995), CMA-ES (Hansen et al., 2003) or L-BFGS (Nocedal, 1980). In the case of Bayesian optimization, the surrogate model uses machine learning to capture previous iterations acting as a memory of the full optimization process. Meanwhile, the decision component carefully selects the next query at each iteration.

In the case of simulation calibration, there are many variables that can be used for fitting, some of them might be even competing. Then, we can redefine the problem as one of multi-objective, multicriteria optimization or Pareto optimization:

(15) $$x^{⋆}=\underset{{}_{x\in \chi }}{\arg\max}(f_{1}(x),f_{2}(x),\ldots ,f_{n}(x)),$$

In this case, the objective is not only to find a single optimal value for the simulator parameters, but to find the whole set of Pareto optimal points, that is, those points that dominate the rest of the points. Although this is a completely different problem, the seminal work of Knowles (2006) extended the Bayesian optimization methodology to the multi-objective setup.

There are several pieces of software that implements Bayesian optimization, like BayesOpt (Martinez-Cantin, 2014). A full review can be found in Shahriari et al. (2016). However, many of them do not support multi-objective optimization and those that do support multiple criteria are very limited in terms of other features. In this work, we have used SigOpt^3^ (Martinez-Cantin et al., 2018) for its support for parallelization and multi-objective optimization. Besides, it provides other features like the parameter importance, which will be discussed in the Results section.

For our experiments, we have decided to fit two competing metrics: the length of the longest protrusion (*llp*) as well as the number of protrusions (*np*) (Figure 1). The fitting of the *in silico* values with respect to the *in vitro* measurements is computed using the Bhattacharyya coefficient (also known as *BC*), which has been widely used to compare the similarity or discriminate of two continuous or discrete distributions (Comaniciu et al., 2000). In fact, for discrimination it corresponds to the upper bound of the Bayesian error when performing Bayesian hypothesis testing with symmetric cost functions and uninformative priors (Nielsen, 2014). Note that, Bayesian hypothesis testing already includes a penalization for model complexity and priors result in a regularization effect, being less sensitive to overfitting than classical hypothesis testing (Kass and Raftery, 1995).

In particular, histograms of both *in vitro* and *in silico* experiments are used as discrete distributions to compute those metrics (Equation 16).

(16) $$\begin{array}{r} BC=\overset{n}{\underset{i=1}{\sum }}\sqrt{hist_{in\;vitro}^{i}\cdot hist_{in\;silico}^{i}}, \end{array}$$

*hist*^*i*^ represents the value of the i-th histogram bin defined as the probability of occurrences of values in the range (*x*_*i*−1_, *x*_*i*_].

The selection of metrics affects model calibration, so we have carefully selected the metrics with a greater influence on cell migration to the best of our knowledge. Moreover, these metrics are based on experimental measurements that we are able to accurately quantify. However, there are other measurements based on cell motion, such as the instant cell speeds, that are so low that we are not able to quantify them with the required accuracy. For those metrics it is only possible to perform a qualitative analysis. Although our proposed metrics are based on just two quantities measured in the experimental data: the length of the longest protrusion and the number of protrusions, we consider that both variables are fundamental in the regulation of the final 3D cell motion. In particular, experimental observations (Moreno-Arotzena et al., 2015; Del Amo et al., 2017; Movilla et al., 2018) suggest that the length of the longest protrusion has great influence over the cell speed whereas the number of protrusions has a great impact on the cell trajectory (whether it is random or directional).

Optimizing the BC function can be considered as a form of Bayesian learning in the sense that we are trying to fit a model that best represents the distribution of the data, and therefore maximizing the posterior. Similarly, optimizing the BC can be seen as a form of Bayesian hypothesis testing where we are rejecting all the models with higher Bayesian error.

Furthermore, Bayesian optimization is a black-box method, meaning that it does not require specific knowledge about the metric, and that metrics can be easily interchanged. Thus, the same methodology can be applied to any other feature or any other similarity metric, such as KL-divergence or any other loss function. Besides, we can include metrics not directly related to the data such as cost, time, etc. These metrics can be competing, meaning that one metric cannot be improved without another metric suffering. As a result, the solutions distributed in the Pareto set might be distributed in a complex way. Thus, sample efficient search like Bayesian optimization is of paramount importance. Besides, the resulting Pareto front allows the expert user to balance the competing metrics a posteriori, choosing the most convenient parametrization in different circumstances.

### Model validation using different chemoattractant concentrations and gradients

After calibrating the numerical model, we have to validate it, testing their predictive ability to simulate different cell responses under different chemical gradients. This validation process allows us to prove that the proposed model does not only accurately replicate the results used to calibrate it, but also new ones, so that there has been no overfitting during the calibration process. In the preceding calibration process, we have used quantitative results related to both the length of the longest protrusion and the number of protrusions of migrating NHDF from *in vitro* experiments without any chemoattractant gradient. However, the validation process of this computational model is based on qualitative observations of migrating cells surrounded by a chemoattractant factor diffusing throughout the ECM (Song et al., 2006; Bosgraaf and Van Haastert, 2009). We have simulated six different extracellular environments. Three of these extracellular environments include different PDGF gradients (10^−1^, 10^0^, 10^1^ μ*M*/*mm*) but a fixed PDGF concentration at the initial cell's position (0.8 μ*M*). The other three extracellular environments include a fixed PDGF gradient (10^0^ μ*M*/*mm*) but different PDGF concentrations at the initial cell's position (0.08, 0.8, and 8.0 μ*M*). Twenty simulations have been executed for each extracellular environment, using the same seeds used during the calibrating process. The comparison between *in vitro* and *in silico* results is based on qualitative observations of the velocity component in the direction of the chemotactic gradient (*v*_*x*_).

We assume a fixed growth factor profile without any induced modifications of the spatial gradient due to the growth factor diffusion throughout the ECM. Thus, the chemoattractant chemical profile is assumed to be temporally stable as the inlets and outlets of our system keep a fixed growth factor profile during our 4-h simulation.

## Results

**Figure 6:**
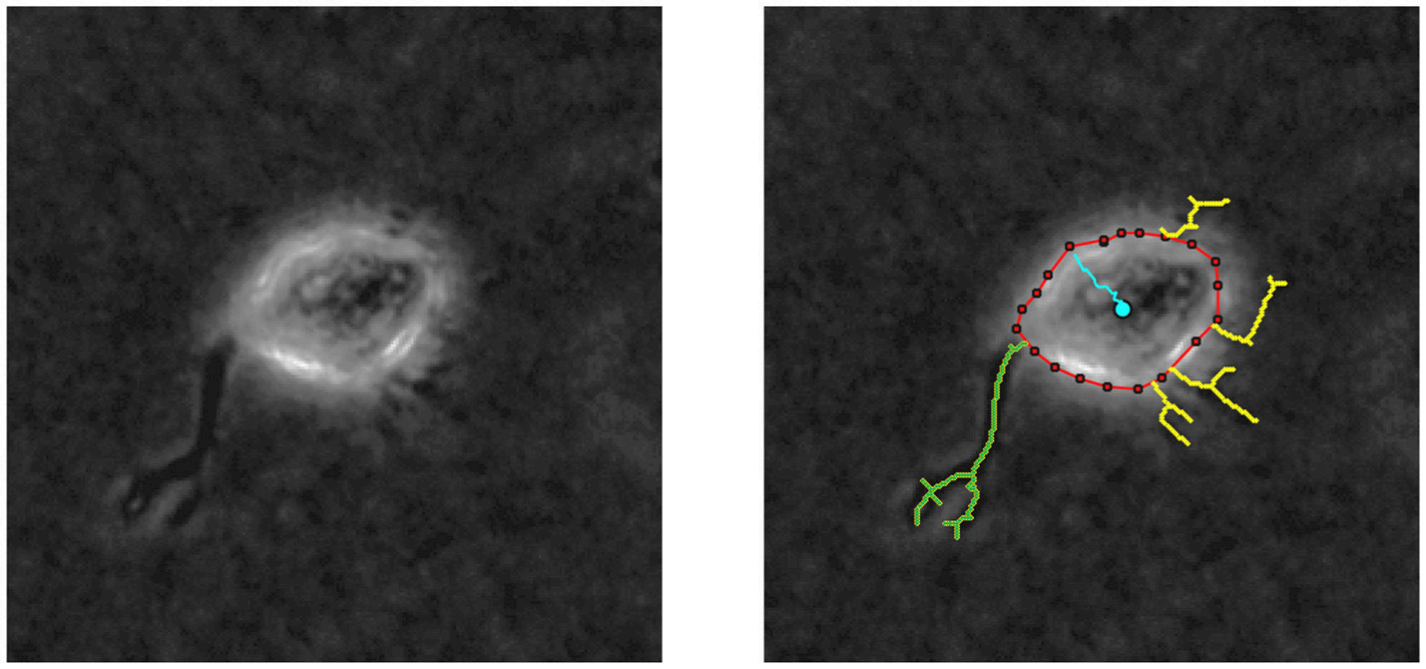
**(Left)** Phase contrast example of a NHDF cell cultivated in a 2 mg/ml collagen gel, with 200x magnification (20x objective) acquired using multidimensional microscopy. **(Right)** Protrusion analysis performed by in-house Matlab algorithms; red line delimits cell body, yellow lines represent the protrusions, and blue line shows cell body displacement. In this case, the longest protrusion is the green one and the number of protrusions is 5.

By means of *in vitro* experiments in fibroblasts, it is possible to quantify both the length of every protrusion, as well as the number of protrusions generated at every checkpoint t (*t* = 0, 5, 10, …, 240 min). Figure 6 shows an example of the images generated by multidimensional microscopy and the posterior protrusions analysis performed using in-house Matlab algorithms. However, our model focuses on the length of only the longest protrusion at each temporal checkpoint *t*, ignoring the length of the other protrusions, as explained in Section Modeling Cell Body Translocation. Therefore, during the calibration process the comparison between *in vitro* and *in silico* experiments is performed by means of the *BC* using these two features (length of the longest protrusion and the number of protrusions generated by migrating cells).

**Figure 7:**
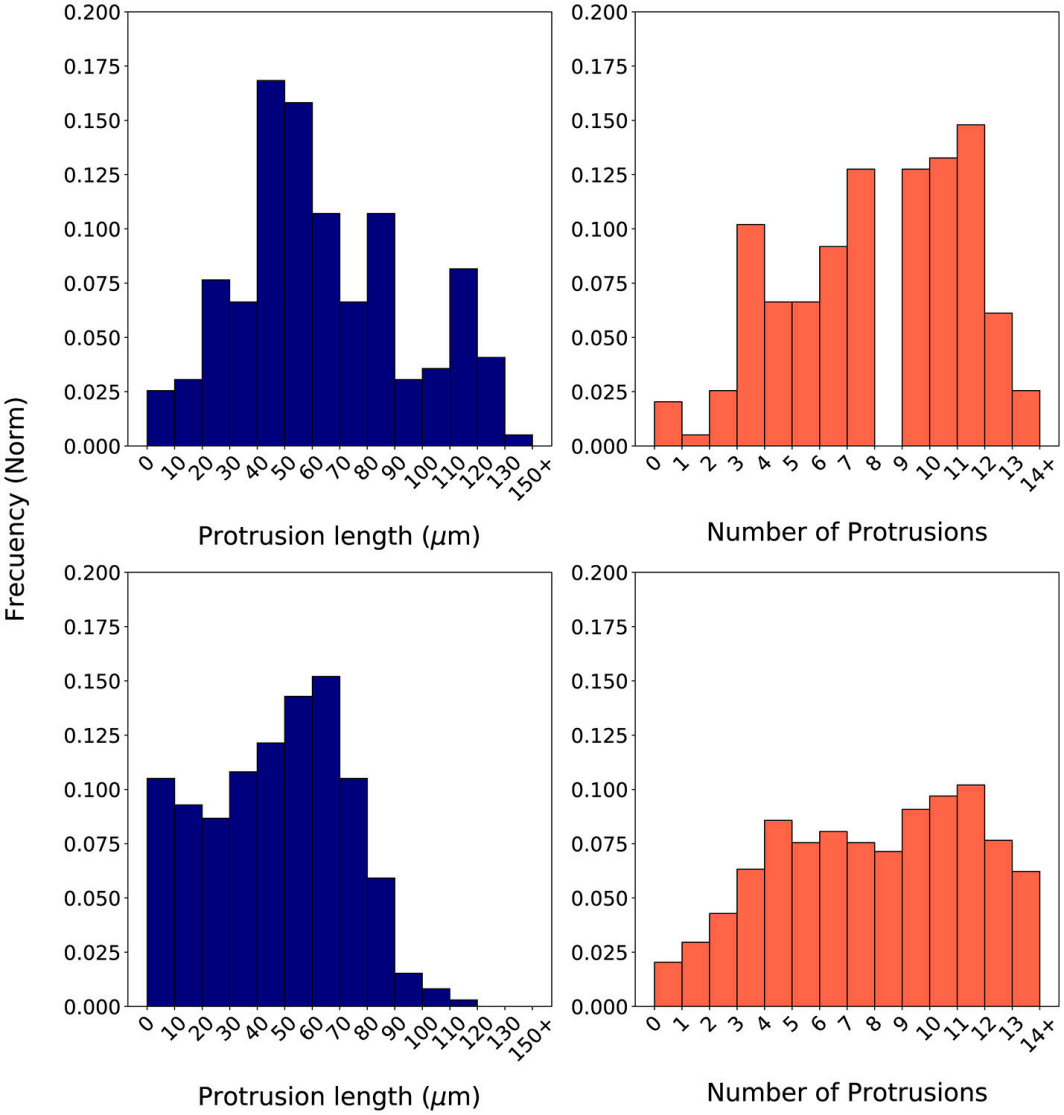
Normalized histograms associated to *in vitro* experiments **(Top)** based on the length of the longest protrusion (measured in μm) **(Left)** and on the number of protrusions **(Right)**. Normalized histograms associated to *in silico* experiments **(Bottom)** based on the length of the longest protrusion (measured in μm) **(Left)** and on the number of protrusions **(Right)**. *In silico* experiments were generated using one of the best parametrizations suggested by SigOpt with metrics *BC*_*llp*_ = 0.87 and *BC*_*np*_ = 0.81.

During calibration, for every iteration in the optimization loop, 20 simulations replicating the *in vitro* scenario of a 2 mg/ml collagen ECM have been executed—in order to capture the stochastic nature of our model. Those 20 simulations used 20 different seeds in order to initialize the global random number generator of our model. Once the 20 simulations have been completed, their associated histograms are computed by means of a computer-based algorithm. These histograms (e.g., Figure 7 bottom) are compared with the *in vitro* histograms (Figure 7 top) using the proposed evaluation metrics *BC*_*llp*_ and *BC*_*np*_ (defined in Equations 18, 19 respectively and based on Equation 16).

(18) $$\begin{array}{ccc} BC_{llp} & = & \frac{\sum_{i=1}^{N}\;BC_{llp}^{i}}{N},\;N=20, \end{array}$$

(19) $$\begin{array}{ccc} BC_{np} & = & \frac{\sum_{i=1}^{N}\;BC_{np}^{i}}{N},\;N=20, \end{array}$$

In order to compute the two metrics using the *BC*, it is required to generate the associated histograms for both the longest protrusion length and the total number of protrusions. Histograms associated to *in vitro* experiments using a cellular microenvironment based on 2 mg/ml collagen gels show how the protrusion length ranges from over 0 μm to almost 140 μm. However, most of the longest protrusions have a length in the interval 40–60 μm (Figure 7 top left). Regarding the number of protrusions, there is a high dispersion, ranging from 1 to 14 protrusions in each individual fibroblast during migration (Figure 7 top right). Figure 7 (bottom) shows an example of a couple of histograms associated to the *in silico* experiments. In this case, we have generated *in silico* histograms using the best parametrization suggested by SigOpt with metrics *BC*_*llp*_ = 0.87 and *BC*_*np*_ = 0.81 (Figure 7 bottom). These histograms show how, although the length of the longest protrusions is between 0 and more than 150 μm, there is a peak in the interval 60–80 μm (Figure 7 bottom left). Regarding the number of protrusions, there are usually about 9 to 12 in each fibroblast during migration (Figure 7 bottom right). When comparing measurements of the length of the longest protrusion, the mean values are 63.71 (*in vitro*) vs. 65.98 (*in silico*), whereas the standard deviations are 31.20 (*in vitro*) vs. 26.82 (*in silico*). For the measurements of the number of protrusions, the mean values are 7.57 (*in vitro*) vs. 7.38 (*in silico*), whereas the standard deviations are 3.27 (*in vitro*) vs. 4.00 (*in silico*).

**Figure 8:**
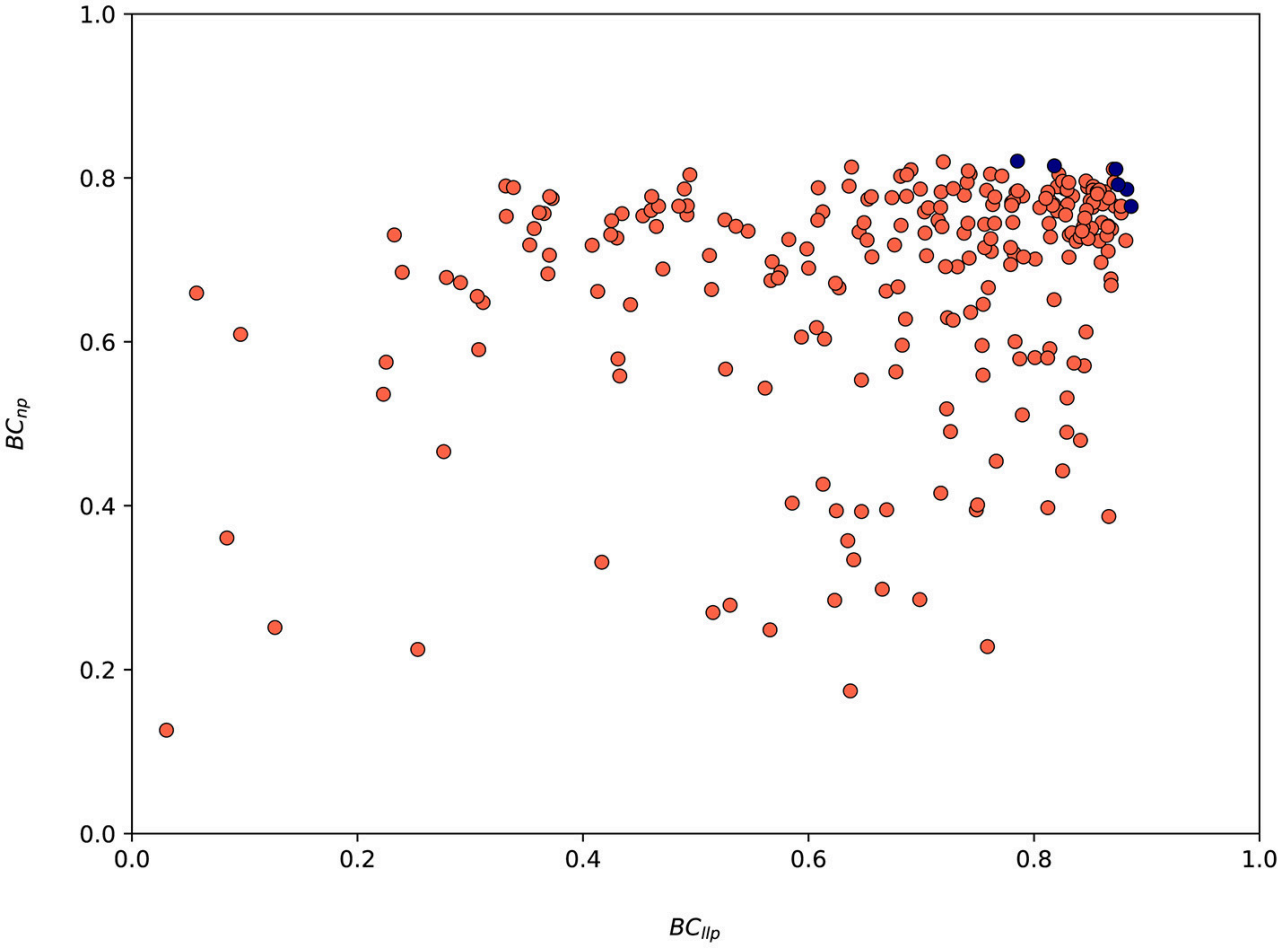
Values associated to both metrics (*BC*_*llp*_ and *BC*_*np*_) for the 300 model parametrizations suggested by SigOpt during the calibration process. Red circles are associated to every parametrization tested whereas the blue ones represent Pareto optimal points (parametrizations where one metric cannot be improved without another metric suffering) and form an approximate Pareto frontier.

The values of both metrics *BC*_*llp*_ and *BC*_*np*_ for every suggested parametrization by SigOpt are shown in Figure 8. SigOpt is able to find parametrizations with higher values of the *BC*_*llp*_ (even higher than 0.9) than the *BC*_*np*_ (always lower than 0.8). Besides, the majority of the parametrizations suggested by SigOpt are higher than 0.7 for both metrics (35.67%), with slightly better results for the metric related with the length of the longest protrusion (Figure 8).

A total of nine parameters of the model have been calibrated (Table 5). The range of values for each parameter has also been established in order to define the search space (Table 5). Note that in this case, the search space includes both continuous regions in the real space and discrete values for integer parameters. Thus becoming a mixed-integer programming problem, much harder to be optimized than just real spaces (non-linear optimization) or integer spaces (combinatorial optimization). For some parameters we have established a range based on the values used in Ribeiro et al. (2017), whereas for others such as *E*_*protrusion*_ we have determined a range based on values found in literature. In addition, for the parameters related to *s* signal (*s*_*birth*_, *s*_*exp*_, *s*_*ret*_, and *s*_*binary*_), we have analyzed the values of *s* at different time steps. These ranges should be biologically relevant. For example, the range of the parameter *E*_*protrusion*_ (protrusions elastic modulus) includes the value given in Mofrad and Kamm (2006) and Li et al. (2014). We have also automatically discarded any parametrization with *s*_*ret*_ ≥ *s*_*birth*_ *or* *s*_*ret*_ ≥ *s*_*exp*_ *or* *s*_exp_ ≥ *s*_*birth*_ because from a biological perspective they are invalid (the minimal amount of signal required for the onset of new protrusions, *s*_*birth*_, and for the reinforcement of pre-existing protrusions, *s*_*exp*_, cannot be lower than the minimal amount of chemotactic signal *s* required to remain active and not disappear, *s*_*ret*_; the minimal amount of signal required for the onset of new protrusions, *s*_*birth*_, cannot be lower than the minimal amount for the reinforcement of pre-existing protrusions either). The parametrization selected as the optimal one after 300 iterations of the calibration process using SigOpt is summarized in Table 2. For example, the best value for the elastic modulus is 10^7^ *Pa*. The best parametrization, with metrics *BC*_*llp*_ = 0.87 and *BC*_*np*_ = 0.81, have been selected due to the balance between both metrics.

**Figure 9:**
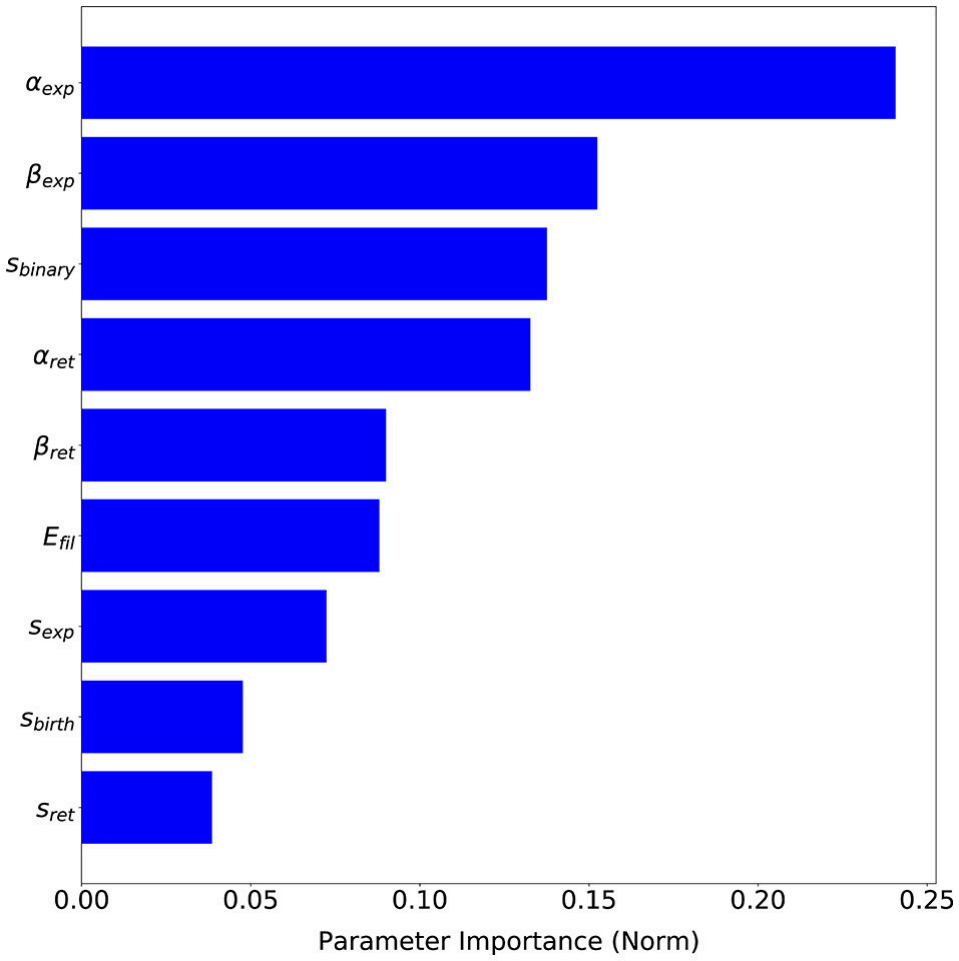
Parameters sensitivity based on SigOpt analysis of each parameter importance on the proposed evaluation metrics.

The advantage of having a probabilistic surrogate model of the metrics is that we can perform other types of data analysis during the optimization process. SigOpt also offers an importance analysis of each parameter on the metrics (see Figure 9), i.e., how influential each parameter is on the metrics, that is, how much the metric values change with variations of each parameter. This analysis gives us valuable insights on our model performance. Although every parameter has some influence over the metrics output, *α*_*exp*_, a parameter which computes the free expansion/retraction stretch rate field (during the protrusion dynamics stage) is the most important parameter (24.06%). The parameters *β*_*exp*_, also used to compute the free expansion/retraction stretch rate field, and *s*_*binary*_, used to simplify the search of signal *s* peaks where protrusions centroids are localized, are the second and third most influential parameters on the metrics (15.25 and 13.76%, respectively). Lastly, *s*_*birth*_ and *s*_*exp*_ are the ones with the least importance on our evaluation metrics (4.76 and 3.86%, respectively).

**Figure 10:**
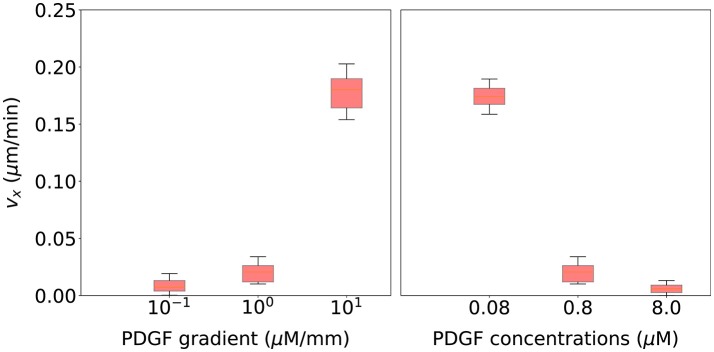
Cell migration speed statistical analysis for 20 simulations using the parametrization selected during the calibration process and associated with six different extracellular environments. **(Left)** three of these extracellular environments include different PDGF gradients (10^−1^, 10^0^, 10^1^ μ*M*/*mm*) but a fixed PDGF concentration at the initial cell's position (0.8 μ*M*). **(Right)** three extracellular environments include a fixed PDGF gradient (10^0^ μ*M*/*mm*) but different PDGF concentrations at the initial cell's position (0.08, 0.8, and 8.0 μ*M*).

Finally, we validate this computational model using qualitative observations based on cell motion of migrating cells surrounded by different chemoattractant gradients. Figure 10 (left) shows that as the PDGF gradient grows, cell's velocity in the direction of the chemotactic gradient increases too. Thus, cells are following a more directional trajectory which agrees with experimental observations from Bosgraaf and Van Haastert (2009). On the other hand, Figure 10 (right) shows that as the PDGF concentration surrounding the cell increases, cell's velocity in the direction of the chemotactic gradient decreases. In this case, cells are following a more random trajectory. This fall in the effective speed of the cell is thought to be associated with receptor saturation (Song et al., 2006).

## Discussion

Understanding the process of cell migration is a really difficult endeavor since it is a biological process coordinated by multiple factors. Temperature (Higazi et al., 1996), adhesion sites in the ECM (Cukierman et al., 2001), ECM mechanical properties and architecture (Wolf et al., 2013) as well as the gradient of chemical factors (Devreotes and Janetopoulos, 2003), modulate cell migration, by regulating the signaling pathways and intracellular cytoskeleton and adhesion organization (Paul et al., 2016).

According to our experimental observations (Moreno-Arotzena et al., 2015; Del Amo et al., 2017; Movilla et al., 2018) cells tend to present two different behaviors: they increase the number of stable protrusions, in which case each protrusion is shorter; or they decrease the number of stable protrusions, in which case at least some of them are longer. In the first case, protrusions compete and there is not any preferential movement. In the second case, normally cells present a defined movement in the direction of the longest protrusion.

Several assumptions are made regarding the mechanical model of the ECM. First, we consider the ECM as an isotropic material. Nevertheless, the ECM is anisotropic due to the different fiber directions (Valero et al., 2018). Second, the mechanical properties of the ECM are assumed homogeneous, thus we do not consider the heterogeneity associated to the distribution of the fibers. Third, ECM remodeling is not considered in this model. However, this is an acceptable approximation for preliminary studies of cell motility in collagen gels, which allows us to use the Eshelby's theory.

Due to the complexity of cell migration, computational models have been widely used to improve its understanding (Rangarajan and Zaman, 2008; Mak et al., 2016; Chen et al., 2017). Cell migration include several stochastic processes such as the evolution of chemically reacting systems. The Stochastic Simulation Algorithm (SSA) (Gillespie, 1976, 1977) has been widely used to numerically simulate the stochastic behavior of biochemical reactions. However, the SSA is considered too slow for many practical applications (Gillespie, 2001, 2007). This effect occurs clearly in our case: even though the SSA offers an exact solution, simulations take too long to finish (an average of 10.77 h of execution time for each simulations of 4 h of cell migration). The tau leaping algorithm has been considered a good fit for our purposes: it gives us a “good-enough” approximation (see footnote 1) of the temporal evolution of our biochemical system and allows us to optimize the numerical performance of our mechanochemical model (an average of 1.28 h of execution time for each simulation of 4 h of cell migration). Thus, reducing the computational cost to almost an order of magnitude.

Although in most computational works (Bauer et al., 2009; Bentley et al., 2009; Vermolen and Javierre, 2012; Daub and Merks, 2013; Talkenberger et al., 2017; Escribano et al., 2018; González-Valverde and García-Aznar, 2018; Kim et al., 2018; Moure and Gomez, 2018) authors perform strong efforts to validate models comparing experimental results with numerical ones, there is a lack of full integration of both kind of results. However, this paper presents a relevant step forward in this direction, showing a novel methodology that integrates both modeling strategies (*in vitro* and *in silico*) by means of the application of Bayesian optimization during the calibration process.

The complexity of the calibration process of any model grows rapidly with the number of parameters. Another factor that greatly increases the complexity of the calibration process is the stochastic nature of some biological models such as the one presented in this paper. Stochastic models require the execution of several simulations for each model parametrization in order to capture the results variation associated to the stochastic randomness. Moreover, if the execution of each simulation takes more than a couple of minutes, a manual approach for this calibration process becomes highly prone to inefficiencies.

When choosing the values for each model parameter using such a manual approach, it is usually the case that researchers turn to literature as their starting point. Then, they perform some manual tuning so simulations results fit approximately the experimental data. Generally, researchers start by modifying just a couple of parameters using some values considered biologically relevant. Then, they analyze how those parameters influence the model output based on the different values tested. They iterate over this process by picking a couple of the remaining parameters in every iteration—ideally, the selected parameters in each iteration are related to each other. This manual approach is really tedious since the modification of some parameters can potentially require the recalibration of some already calibrated parameters. If the model includes a large number of parameters, researchers could start this tuning process by performing a sensitivity analysis (Saltelli, 2002; Bauer et al., 2009; Bentley et al., 2009; Borau et al., 2012; Vermolen and Javierre, 2012; Daub and Merks, 2013; Escribano et al., 2015, 2018; Talkenberger et al., 2017) in order to focus on those parameters with a higher importance on the model output. Due to computational and time restrictions, this manual step does not generally include more than a couple of iterations, even though it is becoming more and more common to have access to a High-Throughput Computing (HTC) environment—which can reduce the required times to run those simulations by parallelizing them.

This paper proposes the application of the Bayesian optimization technique to reduce these inefficiencies. Bayesian optimization, which has been applied to solve a wide range of problems such as machine learning applications (Snoek et al., 2012), robot planning (Martinez-Cantin et al., 2009), simulation design (Brochu et al., 2010), biochemistry (Czarnecki et al., 2015), and dynamical modeling of biological systems (Ulmasov et al., 2016), offers an automated approach for this calibration process. Furthermore, the Bayesian optimization technique is able to minimize the number of parametrizations to test on the computational model and find a good enough fit to *in vitro* observations. In our case, from the 300 different parametrizations tested during the calibration process, only 6 parametrizations (2%) have the two metrics considered (*BC*_*llp*_ and *BC*_*np*_) below 0.5. On the other hand, SigOpt suggests 107 parametrizations (35.67%) with both metrics above 0.7.

Clearly, the methodology here presented—based on the application of Bayesian optimization to compare the results of *in vitro* and *in silico* experiments—has allowed to identify the key parameters that regulates individual 3D fibroblast migration embedded in a collagen-based matrix. In particular, this novel methodology has been applied during the development of a stochastic model that simulates a chemically reacting system based on the biochemical interaction between the PDGF and a specific type of cell surface receptors, the RTKs. This interaction, in turn, triggers a metabolic cascade of internal signaling that activates a cellular chemosensing mechanism. Moreover, the model's calibration has been proven to be a valid and not an overfitted one during the final validation process. In order to validate the selected parametrization, we have simulated cell migration with a diffused chemoattractant factor throughout the ECM and qualitatively compare observations based on cell's velocity in the direction of the chemotactic gradient with results from previous experimental works (Song et al., 2006; Bosgraaf and Van Haastert, 2009). Our results are in agreement with those from *in vitro* experiments, cells follow a more directional motion as the chemoattractant gradient increases. However, when the chemoattractant concentration surrounding the cell reaches a saturation point cells start to lose the ability to sense the chemical cues.

In conclusion, the tau leaping algorithm allows to optimize the performance of stochastic models based on biochemical kinetics by greatly reducing the execution time of its simulations. In addition, by means of Bayesian optimization it is possible to perform model parameters calibration in a very efficient and completely automatic way. As a result, this novel methodology will improve the development of *in silico* models for a better understanding of cell migration.

## Author contributions

FM-C, MG-B, and JG-A designed research; FM-C, MG-B, YJ-L, and JG-A performed research; YJ-L performed *in vitro* experiments, FM-C, MG-B, RM-C, and JG-A analyzed data; FM-C, MG-B, YJ-L, RM-C, and JG-A wrote the paper; FM-C, MG-B, and RM-C defined Bayesian optimization setup and FM-C built a code for the model and performed all simulations.

### Conflict of interest statement

RM-C was employed by company SigOpt, Inc.

The remaining authors declare that the research was conducted in the absence of any commercial or financial relationships that could be construed as a potential conflict of interest.

The reviewer AL and handling editor declared their shared affiliation at the time of the review.

## Footnotes

^1^The “good-enough” expression used here to describe the accuracy of the tau-leaping algorithm comes from previous works such as Lok (2004) where he states that “One acceleration strategy is to abandon absolute mathematical precision in favor of a good-enough approximation. Gillespie has also been a pioneer in this effort. One of his strategies is called ‘tau-leaping'.” This statement is considered valid as long as the leap condition is satisfied, i.e., as long as the probability of each reaction taking place does not change significantly over the time leap.

^2^The leap condition is an accuracy-assuring restriction which states that during the time interval [*t*, *t*+τ] the probability of each reaction channel *R*_*j*_ being fired should remain approximately constant even though all reaction channels may be fired several times.

^3^https://sigopt.com/
