# Supplementary material for: Integration of in vitro and in silico Models Using Bayesian Optimization With an Application to Stochastic Modeling of Mesenchymal 3D Cell Migration
Source: Front Physiol. 2018 Sep 11;9:1246. doi: 10.3389/fphys.2018.01246 (PMC6142046; doi:10.3389/fphys.2018.01246)
Supplement: Supplementary file 1 [file Table_1.DOCX]

Supplementary Material

Integration of in vitro and in silico models using Bayesian optimization with an application to stochastic modeling of mesenchymal 3D cell migration

Francisco Merino-Casallo^1^, María José Gómez-Benito^1^, Yago Juste-Lanas^1^, Ruben Martinez-Cantin^2,3^, José Manuel García-Aznar^1*^

^1^Multiscale in Mechanical and Biological Engineering (M2BE), Aragón Institute of Engineering Research (I3A), Department of Mechanical Engineering, Universidad de Zaragoza, Zaragoza, Spain

^2^Centro Universitario de la Defensa, Zaragoza, Spain

^3^SigOpt, Inc., San Francisco, CA, USA

*** Correspondence:**Dr. José Manuel García-Aznar
jmgaraz@unizar.es

# Supplementary Data

The set of differential equations defining the simplified mathematical model that mimics the chemosensing mechanism of the cell are:

$$\left\{ \begin{aligned} \frac{\partial RTK}{\partial t}=-k_{1}RTK\cdot\left[ F \right]+k_{2}RTKF \left( {ODE}_{1} \right) \\ \frac{\partial RTKF}{\partial t}=k_{1}RTK\cdot\left[ F \right]-k_{2}RTKF \left( {ODE}_{2} \right) \\ \frac{\partial PI3K}{\partial t}=-k_{3}RTKF\cdot PI3K+k_{4}{PI3K}_{A} \left( {ODE}_{3} \right) \\ \frac{\partial{PI3K}_{A}}{\partial t}=k_{3}RTKF\cdot PI3K-k_{4}{PI3K}_{A} \left( {ODE}_{4} \right) \end{aligned} \right.(Eq. S1)$$

For a further explanation see Ribeiro et al. (2017).
